# The Cascade Analysis Tool: software to analyze and optimize care cascades

**DOI:** 10.12688/gatesopenres.13031.2

**Published:** 2019-12-24

**Authors:** David J Kedziora, Romesh Abeysuriya, Cliff C Kerr, George L Chadderdon, Vlad-Ștefan Harbuz, Sarah Metzger, David P Wilson, Robyn M Stuart

**Affiliations:** 1 School of Physics, University of Sydney, Physics Rd, Camperdown, Sydney, NSW 2006, Australia; 2 Institute for Global Health, University College London, 30 Guilford Street, London, WC1N 1EH, UK; 3 Monash University, 553 St Kilda Road, Melbourne, VIC 3004, Australia; 4 Independent researcher, 72 Bayview Street, Prahran, VIC 3181, Australia; 5 Phyramid, Südquaistrasse 14, Kleinhafen, Basel, 4057, Switzerland; 6 Bill and Melinda Gates Foundation, 500 Fifth Avenue North, Seattle, WA, 98109, USA; 7 Department of Mathematical Sciences, University of Copenhagen, Universitetsparken 5, Copenhagen, 2100, Denmark

**Keywords:** cascades, optimization, modeling, service delivery

## Abstract

**Introduction:** Cascades, which track the progressive stages of engagement on the path towards a successful outcome, are increasingly being employed to quantitatively assess progress towards targets associated with health and development responses. Maximizing the proportion of people with successful outcomes within a budget-constrained context requires identifying and implementing interventions that are not only effective, but also cost-effective.

**Methods:** We developed a software application called the Cascade Analysis Tool that implements advanced analysis and optimization methods for understanding cascades, combined with the flexibility to enable application across a wide range of areas in health and development. The tool allows users to design the cascade, collate and enter data, and then use the built-in analysis methods in order to answer key policy questions, such as: understanding where the biggest drop-offs along the cascade are; visualizing how the cascade varies by population; investigating the impact of introducing a new intervention or scaling up/down existing interventions; and estimating how available funding should be optimally allocated among available interventions in order to achieve a variety of different objectives selectable by the user (such as optimizing cascade outcomes in target years). The Cascade Analysis Tool is available via a user-friendly web-based application, and comes with a user guide, a library of pre-made examples, and training materials.

**Discussion:** Whilst the Cascade Analysis Tool is still in the early stages of existence, it has already shown promise in preliminary applications, and we believe there is potential for it to help make sense of the increasing quantities of data on cascades.

## Introduction

The pursuit of effective program delivery has become a dominant theme in the discussions and strategic thinking of both national and international health and development agencies. Both the Paris Declaration on Aid Effectiveness ^1^ and the Accra Agenda for Action ^2^ emphasized the need for results-based evaluation to assess whether funds are being used efficiently towards achieving desired outcomes, and this has played an important role in shaping the thinking around results measurement more broadly. To support the emphasis on results-based evaluation, a multitude of systems are in operation for collecting and aggregating program result data ^3^. In theory, these data are intended to enable organizations to assess which strategies and programs are effective, identify elements of programs associated with better results, demonstrate accountability to external stakeholders, and make decisions about allocating further funding ^3^. In practice, however, there is a disconnect between the data being collected and the methods available for analyzing them.

**Figure 1.**
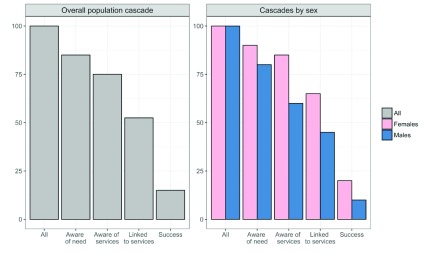
A typical cascade presents the proportion of the total target population that have attained each of the sequential steps of engagement in the path towards a successful outcome.

One method for quantifying how health and development programs are servicing the needs of communities is to define progressive stages of engagement on the path towards a successful outcome, and to measure what proportion of the overall target population has attained each stage. Often, these proportions are plotted as successive bars, in a representation known as a *cascade*, a *care cascade,* a *continuum of care*, or a *service cascade* ( Figure 1). Cascades can be studied at a population level (left panel of Figure 1), or at a disaggregated sub-population level (right panel of Figure 1). In recognition of their importance in understanding health quality, the 2018 Lancet Global Health Commission on High-Quality Health Systems argued that care cascade analyses should be a central component of health quality dashboards for understanding quality of care ^4^.

The left panel is aggregated across the total population, and the right panel shows the same information but disaggregated by sex.

Within public health, cascade-type models were explored as early as the 1960s for analyzing the success of tuberculosis programs ^5^, but the concept of a cascade really gained traction within HIV ^6^, where it is used to characterize the steps of care that people living with HIV go through. The HIV care cascade has been adopted in many countries as a population-level tool to evaluate the progress of individuals through the HIV care continuum ^6– 9^. Following their success in HIV, cascades began to be applied to other areas of health. Tuberculosis followed soon after HIV, with the 2014 End TB Strategy including targets related to the latent tuberculosis cascade of care, and the following year’s Global Plan To End TB 2016–2020 ^10^ including a commitment to measure progress towards these targets. Subsequently, an explicit framework of analysis to account for the losses during each individual step in this cascade was developed for latent TB ^11^ and applied in South Africa ^12^, India ^13^ and many other countries (see also 14 for a methodological framework for active TB disease). The cascade approach has also been applied to the analysis of diabetes, most notably in a 2014 study that provided a comprehensive overview of the continuum of U.S. diabetes care (including a visualization of gaps in awareness of diagnosis, engagement, and treatment) by analyzing nationally representative data benchmarked against care recommendations for cardiovascular risk management ^15^. Building on this, a recent study in South Africa used data from the first comprehensive national survey on non-communicable diseases to construct a diabetes care cascade by categorizing the population with diabetes into those who were unscreened, screened but undiagnosed, diagnosed but untreated, treated but uncontrolled, and treated and controlled ^16^. The cascade framework has also been proposed as an analytic tool in hepatitis C ^17^, other sexually transmitted infections ^18, 19^, addiction care ^20, 21^, and mental health ^22^. Outside of public health, a related concept – funnel analyses – have proven useful in analyzing consumer behavior within ecommerce, retail, and online gaming/applications.

Across all of these different applications, cascades have proven to be an effective visual tool for identifying weaknesses at different stages of service engagement, as well as unacceptable variations between different groups or countries. In addition, a handful of studies have pushed the analytic capacity of cascades one step further, employing them as a tool for identifying what mix of technologies and services should be provided, and to which populations, in order to best ensure that outcomes are met. A study of the HIV cascade in Kenya looked at how varying the coverage levels of five different interventions could improve the care cascade ^23^. An unpublished study conducted in South Africa further extended this idea, introducing the concept of ‘optimizing the cascade’, which meant calculating the coverage levels across 30+ HIV interventions that would maximize the number of people virally suppressed by 2030.

Although there are many prior examples of cascade analyses, and even a few specifically on cascade optimization, these have all been disease-specific use cases. The lack of a readily-available modelling tool has substantially limited the potential for widespread uptake of cascade analyses and cascade optimizations. The purpose of this work is to begin with the concept of a cascade and implement it as a general software tool – called the Cascade Analysis Tool – that allows the same quantitative methods to be applied across application areas in health and development. In many real-world situations, the impact of changing intervention coverage and the way in which interventions should be prioritized is not clear from an analysis of the intervention properties alone. The Cascade Analysis Tool allows users to construct scenarios and optimizations in order to quantitatively answer questions about intervention effects and priorities. Scenarios can be used within the Cascade Analysis Tool in order to assess the impact on the cascade of varying the investment or coverage level of a given intervention or modality. Although scenarios are useful for analyzing cascades and for gaining insight on the impact of scaling up or down particular interventions or modalities, in realistic settings there are a very large number of different possibilities, and it quickly becomes infeasible to rely on constructing scenarios in order to determine what investment priorities should be. This is especially difficult given that the optimal investment strategy may change from year to year. For example, it might be optimal to start by scaling up treatment initiation services until everyone in need of treatment can access it, and then to focus investments on adherence and retention strategies subsequently.

**Figure 2.**
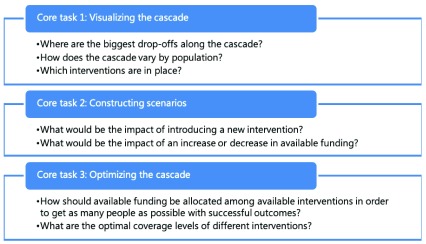
The types of questions designed to be answered by the Cascade Analysis Tool.

The Cascade Analysis Tool is intended to address a set of key policy questions, as outlined in Figure 2. Methodologically, it is based on a compartmental mathematical model structure equipped with methods for parameterizing transition probabilities, and with a suite of inbuilt optimization methods for ‘optimizing the cascade’; that is, finding the annual investment or coverage levels for each intervention that would result in the cascade being as close as possible to some target distribution, subject to constraints on the overall budget and the pace of scale-up over time.

The Cascade Analysis Tool is an open access software package, accessible via a web-based application. Throughout this paper, we refer to an illustrative example of a hypertension cascade; this example, along with several other pre-made models, are available to all users as part of the library of ‘demo’ projects included with the software.

The Cascade Analysis Tool is intended to provide a practical way for stakeholders to utilize the increasing quantities of data on the costs, coverage, and impact of health and development interventions, and modalities through which these interventions are delivered to individuals, thus addressing some of the disconnect between the kinds of data being collected and the methods available for analyzing them.

## Methods

### Implementation

**Figure 3.**
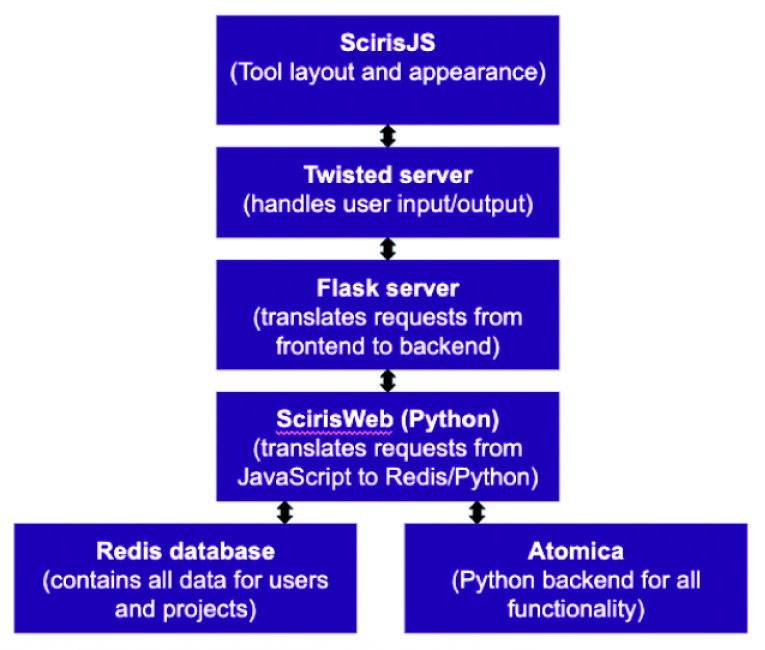
The software architecture of the Cascade Analysis Tool.

The Cascade Analysis Tool is a web application, compatible with any browser, that provides a user-friendly interface for designing and analyzing care cascades. The backend is powered by Atomica (a Python package for making and analyzing compartmental models), the web application is built in Python with ScirisWeb, and the frontend is built in JavaScript with ScirisJS ( Figure 3). Although intended to run on cloud servers, it can also be run on a personal computer operating Windows, MacOS, or Linux.

**Figure 4.**
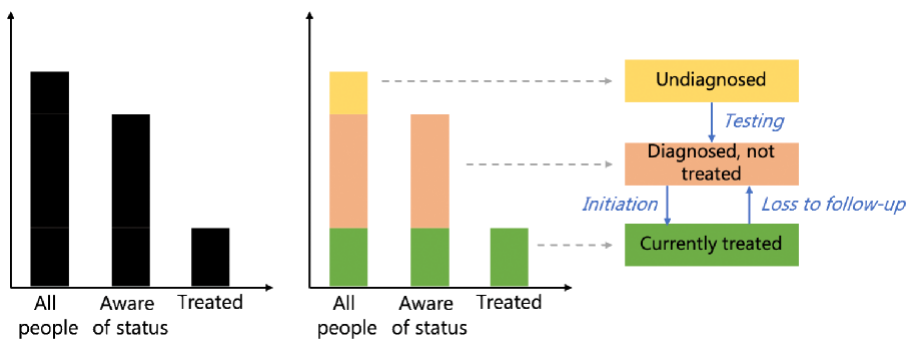
A generic treatment cascade (panel A), which is translated (panel B) into a compartmental model (panel C).

The functionality for running cascade analyses with the tool relies on Atomica’s flexibility for creating general compartmental models with arbitrary compartments and transitions. With the Cascade Analysis Tool, users can define how different compartments are combined into cascade stages ( Figure 4), so that the tool can be used to project how a care cascade will evolve over time. Conceptually, arbitrary cascades are created following the process depicted in Figure 4. Beginning with a simple cascade representation, in which the progressive stages along the path to a successful outcome are plotted ( Figure 4a), the next step is to break down each cascade bar so that it consists of the sum of all the bars that came before it, plus the difference between the height of the previous bars and the height of the current bar ( Figure 4b). These differences represent the mutually exclusive states that a person can be in. This representation in terms of mutually exclusive states allows us to model the cascade using a compartmental model ( Figure 4c), which is carried out in the Atomica package.

### Operation

The workflow for creating and analyzing a cascade in the Cascade Analysis Tool consists of three key steps: designing the cascade (optional), collating data, and analysis. If using one of the pre-made cascades in the Cascade Analysis Tool’s library, it is possible to skip the first step.

***Designing the cascade.*** All of the information about the design of the cascade is entered in a *framework file*, which is an Excel template that can be uploaded through the software. This file contains the specifications of the compartmental model that is used to construct the cascade, including the compartments, transitions, parameters, and derived metrics (e.g. cascade stages). For example, to set up the model described in Figure 4, users would define 3 compartments (undiagnosed, diagnosed not on treatment, and currently treated) and 3 transitions (testing, initiation, and loss to follow-up). Within the Cascade Analysis Tool, each transition is given a name and a definition in terms of function of one or more parameters. ** A parameter can be associated with more than one transition – for example, the annual probability of death applies to all individuals regardless of their disease status. When using the Cascade Analysis Tool, users have a choice of either directly entering data on these parameters, or allowing their values to be calculated as a function of other model quantities by entering formulas into the framework file. This means that complex computations and functional dependencies can be readily used. Finally, users specify the cascade stages and any other derived metrics of the compartmental model (e.g. in Figure 4, the cascade stage “Diagnosed” would consist of the sum of “Diagnosed, not treated” and “Currently treated”).

Given the flexibility of defining a cascade based on a compartmental model, it is possible to specify multiple different “types” of cascade using the Cascade Analysis Tool. This includes cascades where it is possible for people to move forwards and backwards through the cascade stages (e.g., with HIV, people may move from “successfully treated” to “on treatment but with poor outcomes”), as well as cascades where people who are not successfully treated move back to the beginning of the cascade.

### Data entry

The next key step in a cascade analysis is to collate and enter data. This is specific to a particular context; users create a *project* for encapsulating all of the data and analyses specific to that context. Creating a project requires selecting the framework that will be used as the basis for the cascade model structure, selecting the number of populations to include, and selecting the years for which data will be collected. Data entry itself is done in two Excel spreadsheets, referred to as the *databook* and the *program book*, both of which are automatically created by the Cascade Analysis Tool once a project has been created. Within the databook, users enter data on each parameter that influences transitions through the cascade, and within the program book, they enter data on the interventions that influence the parameters. The Cascade Analysis Tool comes with a library of pre-filled databooks and program books that can be immediately used for demonstration analyses.

As with any compartmental model, the minimal data requirements for running a cascade analysis include (a) initial conditions on the number of people in each compartment, and (b) data/estimates to inform the transitions between compartments. For example, the model described in Figure 4 could be set up with data/estimates on the number of people in each of the 3 compartments at a single point in time, plus data/estimates on testing, initiation, and loss to follow-up (e.g. annual number tested/initiated/lost, annual probability of testing/initiation/loss, or proportion of individuals that tested/initiated/were lost within the last 12 months). In addition, modelling the effects of interventions requires data/estimates on (a) the unit costs of each intervention, (b) the current coverage of each intervention, and (c) for certain interventions, the efficacy of the intervention, i.e. how it influences model parameters. Continuing the example in Figure 4, if we know that the unit costs of testing, initiation, and adherence programs are $10, $18, and $30, respectively, and that there were 1000 people tested, 800 initiated onto treatment, and 300 enrolled in adherence programs in a given year, then we could enter these data into the program book in order to run a cascade analysis. To understand the effects of the adherence program, we would also need to specify that being enrolled in the adherence programs reduces the probability that an individual is lost to follow-up by a certain amount.

Typical sources of inputs for the Cascade Analysis Tool may include: Demographic and Health Surveys (DHS); the Institute of Health Metrics Evaluation (IHME) for estimates of the burden of disease; the Global Health Costing Consortium (GHCC) for data on the unit costs of interventions; in-country studies of the efficacy and costs of interventions; academic studies on the clinical efficacy of biomedical interventions; and in-country studies of cascade dynamics.

### Analysis of policy questions

Having completed the cascade design and gathered the data, it is possible to begin using the framework to analyze policy questions, such as those illustrated in Figure 2.

The Cascade Analysis Tool contains a set of inbuilt optimization functions that can calculate the distribution of funding across service delivery modalities that results in the best possible cascade. ‘Best’ can be defined by the user – often, the aim is for as many people as possible to attain a successful outcome; in this case, the optimization algorithm would calculate the mix of investments that maximizes the proportion of the population with successful outcomes. However, it is also possible to specify different strategic goals, such as maximizing the number of people diagnosed. This functionality within the tool is primarily intended for central decision makers who are choosing the allocation of a budget. Fundamentally, the optimization system in the Cascade Analysis Tool seeks to modify the timing and funding allocation of interventions to optimize an aspect of the model outputs, subject to constraints on the changes it is allowed to make. 

The optimization problem is separated into two components

- The objective (i.e. defining what we are trying to achieve, and by when)
- The adjustment(s) (i.e., what can be adjusted, and when, in order to meet the objectives)
- Constraints (i.e., what conditions must be satisfied)

Separating these components out means they can be mixed-and-matched to suit a specific optimization problem. Finally, the optimization is numerically performed using one of several algorithms selectable by the user.

*Objectives (i.e., what are we trying to achieve, and by when)?*

The Cascade Analysis Tool supports the following default options:

- Minimize the total number of people lost from each stage of the cascade
- Maximize the number of people at any given stage of the cascade
- Minimize the amount of funding required to meet a certain cascade target

Atomica, the model underlying the Cascade Analysis Tool, has greater flexibility and allows users to construct their own objective using any of the model’s outputs, as well as to combine multiple objectives into a single target. However, this is not currently supported in the Cascade Analysis Tool web application.

*Adjustment type (i.e., what can be adjusted, and when, in order to meet the objectives)?*

The adjustments for an optimization are a specification of what is allowed to be changed in the model in order to achieve the optimal result. The Cascade Analysis Tool has several default options for possible adjustments:

- Immediate one-off allocation change: we ask what share of the annual budget should be allocated to each intervention in order to meet the objectives, subject to any constraints (see “Constraints” section below). The share of the budget allocated to each intervention is assumed to be constant over time, and we assume that the allocation of the budget can change immediately.
- Delayed one-off allocation change: we ask what share of the annual budget should be allocated to each intervention in order to meet the objectives, subject to any constraints. The share of the budget allocated to each intervention is assumed to be constant over time, and we assume that the allocation of the budget can only change after a given year (for example, perhaps change can only take effect in the next planning phase).
- Ongoing (time-varying) allocation changes: we ask what share of the annual budget should be allocated to each intervention in order to meet the objectives, subject to any constraints. The share of the budget allocated to each intervention is allowed to vary over time, according to a schedule defined by the user (for example, it may be possible to change the allocation every year, or every three years, etc.).
- Start-year optimization: rather than varying the share of the budget allocated to each intervention, in this case we seek the optimal timing of making a budget reallocation, subject to any constraints.

*Constraints (i.e., what conditions must be satisfied)?*

Constraints limit possible options when optimizing. They serve as requirements that must be met by any proposed solution. The Cascade Analysis Tool has two principal types of constraint:

- Constraints on individual adjustments: These typically set minimum or maximum amounts of funding that can be allocated to each intervention independently. These may be constant, or they may vary over time when optimizing scale-up or scale-down scenarios.
- Constraining the total budget: there is an overall fixed budget, which is either assumed to be constant over time, or allowed to vary over time (e.g., annually).

*Optimization algorithms*

After defining the optimization, the tool produces an objective function that can be used to perform the numerical optimization using one of several different algorithms. The Cascade Analysis Tool has built-in support for the following algorithms:

- Adaptive Stochastic Descent (ASD) implemented by the *sciris* Python package. This is a gradient-descent type optimizer that performs well at finding local minima ^24^.
- Particle swarm optimization (PSO) implemented by the *pyswarm* Python package. This algorithm is computationally expensive but is more robust than ASD in the presence of multiple local-minima.
- Bayesian Optimization implemented by the *hyperopt* Python package. This method balances exploration of global and local minima, and it is designed to work with expensive objective functions. It is less computationally expensive than PSO and is likely to locate the correct local minimum faster than ASD, although after finding it, it is typically slower to converge to the final optimal solution.

The design of the optimization system facilitates its use with general third-party optimization packages, which makes it easy to switch algorithms and compare different algorithms depending on their suitability to the specific problem at hand.

## Use cases

To illustrate the process of creating a model in the Cascade Analysis Tool, we will construct a hypertension cascade. This cascade is included in the library of demonstration projects available in the Cascade Analysis Tool.

### Designing the cascade

To begin, we need to define the structure of the hypertension model. We consider four disease stages – undiagnosed, diagnosed, on treatment, and successfully controlled.

Next, we consider the possible transitions in the model. People begin in the undiagnosed compartment, and then they progress sequentially through the compartments. Once an individual is diagnosed, they cannot lose their diagnosed status, so there is no transition from diagnosed back to undiagnosed. However, an individual on treatment (or with successfully controlled hypertension) may discontinue treatment, so we include a transition from treatment/controlled back to diagnosed to account for this. An individual may die at any stage, so all compartments also have outflows associated with death – typically, the net death rate would be higher for compartments where individuals have untreated hypertension.

### Data entry

**Figure 5.**
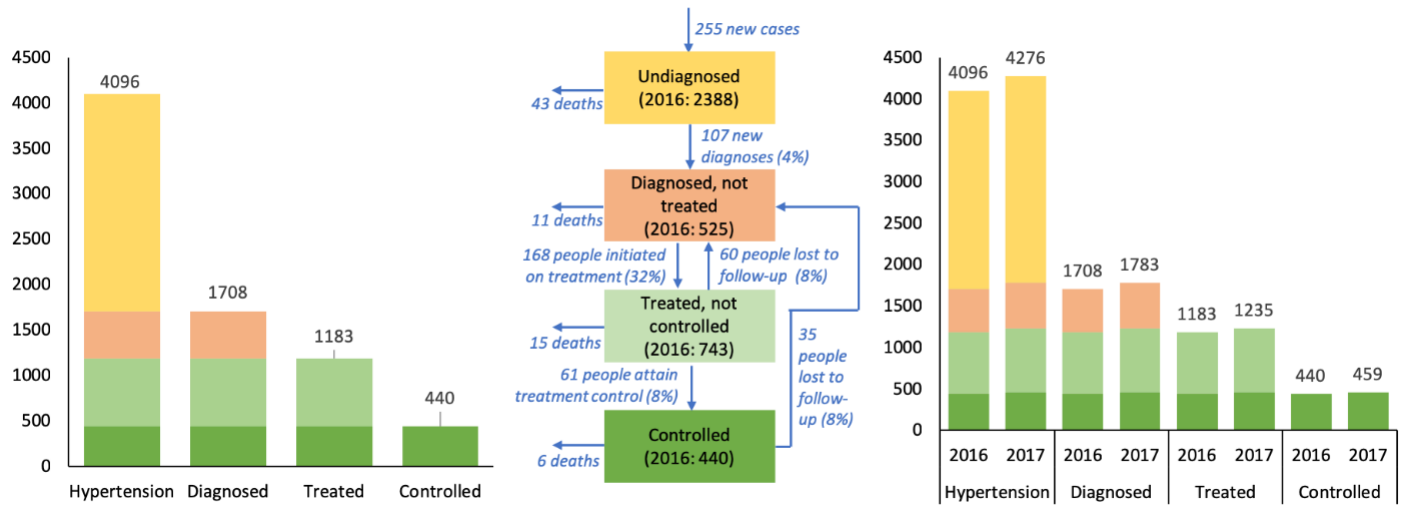
(Panel A) Input data on the state of the hypertension cascade; (Panel b) Illustrative hypertension cascade model with flow rates described in blue text; (Panel C) Cascade representation of the hypertension model with the 2016 values as per the input data and the 2017 values derived from applying the flow rates in Panel B.

***Data entry in the databook.*** We construct a hypothetical example loosely based on a study of 28891 adults in Malawi conducted between May 16, 2013, and Feb 8, 2016 ^25^, which identified 4096 people with hypertension, of which 1708 were aware of their status, 1183 were receiving treatment, and 440 had controlled blood pressure. We assume these numbers describe the state of the hypertension cascade in 2016 ( Figure 5a), and that we want to estimate the state of the cascade in 2017.

**Figure 6.**
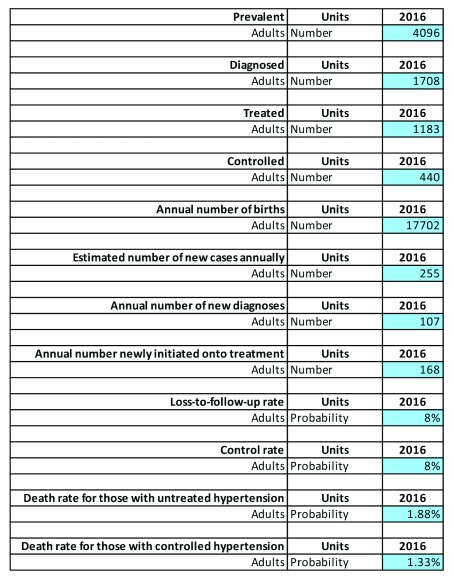
Illustration of the data entry book for the hypertension example depicted in Figure 5.

For flow rates, we assume incidence of 72 per 1000 person-years (averaging the values for reported in 26, 27), which gives 255 new cases/year. Next, we use a mortality estimate of 18.8/1000, reduced to 13.3/1000 for those with blood pressure control (taken from 28), which implies 75 deaths annually among the study population. Combining these estimates of incidence and mortality implies that the total number of people with hypertension increases by 255-75=180 annually, or 4.4%, consistent with an increasing epidemic. We then make additional assumptions on the annual number of people newly diagnosed, initiated on treatment, attaining treatment control, and lost to follow-up, indicated in Figure 5 and Figure 6. This allows us to predict hypertension care cascade stages over time and to estimate the number of people in each cascade stage in 2017, depicted in Figure 5. These data and assumptions are entered into the databook ( Figure 6).

Although we have illustrated values for a single year in this example, the databook supports entering values at multiple time points. Complex models may have many more compartments and parameters, and we have tested the software with highly complex models with ~ 30 compartments and ~150 parameters to verify scalability.

***Data entry in the program book.*** One of the key purposes of a cascade analysis is to understand how various different interventions affect the state of the cascade. In our hypertension cascade example, it would be reasonable to expect that several of the variables that affected the movement of people through the cascade are dependent on interventions that determine the testing, treatment initiation, treatment success, and loss-to follow-up rates.

An essential part of the data collation and curation stage involves assembling a list of the interventions that are likely to have an impact on the cascade. We now illustrate how the programmatic data are used by continuing the hypertension example, supplemented with some assumptions on programmatic data.

We will suppose that people are diagnosed with hypertension after receiving blood pressure tests, and that 2580 such tests were conducted in 2016, either through pharmacies (which we assume provide 55% of tests at a unit cost of $5 and with yield of 3.5%), in clinics (which we assume provide 40% of tests at a unit cost of $20 and with yield of 3.5%), or via an outreach program (which we assume provide the remaining 5% of tests at a unit cost of $15 and with yield of 15%). We suppose that people are initiated onto treatment either immediately after diagnosis (with 20% of those diagnosed at pharmacies, 90% of those diagnosed in clinics, and 70% of those diagnosed via outreach programs being immediately initiated onto treatment), or else people are offered treatment and lifestyle counseling at a unit cost of $25. We also suppose that there is an adherence and lifestyle counseling program to assist those on treatment without blood pressure control (operating at a unit cost of $25 and with 30% of those counseled attaining blood pressure control within 3 months), and retention enhancement initiatives (such as automatic prescription refills, text message reminders for taking medication, or dietary support programs) to counteract loss to follow-up, which increase treatment retention from 88% to 96% at a unit cost per person counseled of $25. Based on what we know about the flow rates through the cascade from Figure 5 and these assumptions about hypothetical programmatic effects, we obtain the programmatic summary documented in Table 1.

**Table 1.** Illustrative data on interventions for the hypertension example presented in Figure 4.

Intervention	Target cascade stage	Number covered	Unit cost	Baseline investment *	Impact
Pharmacy testing	Undiagnosed	1,430 tested	$5	$7,150	• 50 diagnosed (3.5% yield) • 20% start treatment (10 people)
Clinic testing	Undiagnosed	1,000 tested	$20	$20,000	• 35 diagnosed (3.5% yield) • 90% start treatment (32 people)
Outreach testing	Undiagnosed	150 tested	$15	$2,250	• 22 diagnosed (15% yield) • 70% start treatment (16 people)
Treatment & lifestyle counseling	Diagnosed, not treated	110 counseled	$25	$2,750	• All those counseled start treatment (110 people)
Adherence & lifestyle counseling	Treated, not controlled	200 counseled	$25	$5,000	• 30% of those counseled attain blood pressure control within 3 months (60 people)
Retention enhancement initiatives	All treated	600 covered	$25	$15,000	• 96% treatment retention vs 88% among those not covered (95 people lost)

*Baseline investment is calculated here by multiplying the number of people covered by the unit cost.

### Analysis of policy questions

**Figure 7.**
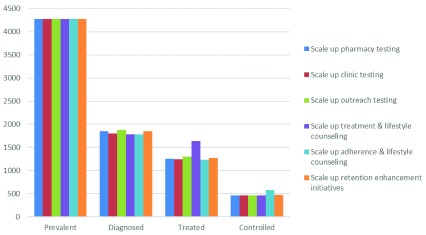
The state of the illustrative hypertension cascade in 2017 under the 6 different scale-up options presented in Table 2.

***Case scenarios.*** To illustrate the use of scenarios, we consider six different scenarios in which an additional $10,000 is allocated to each of the six interventions indicated in Table 1 and calculate the impact that this would have on the hypertension cascade introduced in Figure 5. These scenarios are presented in Table 2 and Figure 7.

**Table 2.** Illustrative scenarios showing different intervention scale-up options, based on the treatment cascade introduced in Figure 3.

Intervention to scale up	Scaled-up budget	Number covered	Impact	Cascade in 2017 (% improvement over baseline)
Pharmacy testing	$17,150	3,430 tested	• 120 diagnosed • 24 start treatment	• Diagnosed: 1853 (4%) • Treated: 1249 (1%) • Controlled: 459 (-)
Clinic testing	$30,000	1,500 tested	• 53 diagnosed • 48 start treatment	• Diagnosed: 1800 (1%) • Treated: 1247 (1%) • Controlled: 459 (-)
Outreach testing	$12,250	817 tested	• 122 diagnosed • 86 start treatment	• Diagnosed: 1883 (6%) • Treated: 1305 (6%) • Controlled: 459 (-)
Treatment & lifestyle counseling	$12,750	510 counseled	• 510 start treatment	• Diagnosed: 1783 (-) • Treated: 1635 (32%) • Controlled: 459 (-)
Adherence & lifestyle counseling	$15,000	600 counseled	• 180 attain control	• Diagnosed: 1783 (-) • Treated: 1235 (-) • Controlled: 579 (26%)
Retention enhancement initiatives	$25,000	1000 covered	• 62 lost to follow-up	• Diagnosed: 1853 (-) • Treated: 1268 (3%) • Controlled: 472 (3%)

**Figure 8.**
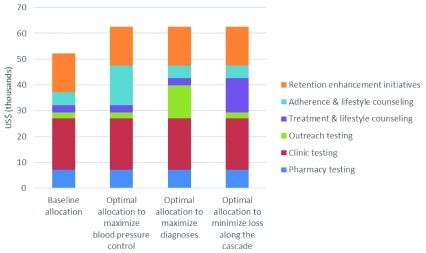
Optimal allocations for achieving difference targets related to the illustrative hypertension example.

***Optimization.*** To illustrate the concept of cascade optimization, we continue our hypertension example, where we have an additional $10,000 to improve some cascade outcome. Figure 8 indicates that the best way to spend these additional funds depends on the objective: if we want to maximize the number of people with blood pressure control, the highest priorities are to scale up the adherence & lifestyle counseling program; if we want to maximize the number of people diagnosed, then the outreach testing program is prioritized; and if we want to minimize losses across the whole cascade, the treatment & lifestyle counseling program is prioritized. Here, the *adjustment type* falls under the heading “immediate one-off allocation change”, since we wish to immediately allocate the additional funds and there are no defined constraints.

We provide three additional examples of optimization problems:

1. The national government is trying to determine an optimal investment strategy for the HIV response in order to get as close as possible to the target of having 86% of people virally suppressed by 2030 (in line with international targets of having 95% of people with HIV diagnosed, 95% of those diagnosed receiving treatment, and 95% of those treated virally suppressed). The country’s treatment program is currently funded by international donors, who have already announced their investment strategy and will begin gradually defunding the treatment program starting in 2021. The government is committed to providing ongoing care for those already on treatment, so the government’s allocation to the treatment will need to increase to match current levels. In this case:
  a. the *objective* is to maximize the number of people in the final stage of the cascade;
  b. the *adjustment type* falls under the heading “ongoing (time-varying) allocation changes”, since the government can change the allocation of funding for the HIV response annually between now and 2030; and,
  c. the *constraints* are the overall budget in each year, plus the additional constraint that the allocation to the treatment program needs to match current levels after international funders have withdrawn.
2. The national government wants to run a large-scale diabetes screening campaign to get 100,000 people screened within the next year. There are several different service delivery modalities for the screening program (e.g., screening through primary health clinics, workplace programs, community outreach programs, and pharmacies). In this case:
  a. the *objective* is to minimize the budget required to attain the target of 100,000 people screened;
  b. the *adjustment type* falls under the heading “immediate one-off allocation change”; and,
  c. there are no defined constraints.
3. The government is considering a program, to be launched in 2022, to improve the overall cascade of care for pregnant women. In this case:
  a. the *objective* might be to minimize losses along the cascade;
  b. the *adjustment type* falls under the heading delayed one-off allocation change”; and,
  c. there may be an overall budget constraint, or other defined constraints such as ensuring minimum funding levels for other programs. For example, it could be specified that funding for the new program can only be taken from new funds plus partial redirection of resources from certain programs while not changing funding for others.

As seen in these examples, the objectives, adjustables, constraints, and optimization algorithms can be flexibly combined by users of the Cascade Analysis Tool.

## Discussion

For complex cascades, it is difficult to determine which programs have the greatest marginal impact. This is especially true when interventions do not target the same populations, do not have the same type of effects, and/or do not have simple linear cost functions. In many cases, the impact of a resource allocation on the cascade may not be known *a priori*. Moreover, when a large number of interventions are involved, the combinatorial explosion of possible budgets makes it computationally infeasible to explore different possible funding combinations using an undirected approach. Previous studies have already shown that targeting investment to the right combination of effective service delivery modalities across the cascade can lead to greatly improved outcomes ^23, 29^. The Cascade Analysis Tool can help make practical recommendations for how to improve cascade outcomes by making use of the increasing quantities of available raw data on the costs, coverage, and impact of health and development interventions. Given the generality of the approach, there is potential for gains to be identified across any number of application areas.

We have taken several steps to encourage the adoption of our framework for cascade analysis. Firstly, we have implemented the processes for using the generalized framework in an open access software package, developed in Python and available via GitHub. Secondly, we have included several simple pre-designed cascades in the software package. Thirdly, we have developed a graphical user interface, a website with additional information, a comprehensive user guide, and a feedback page (also accessible via the tool) where users can provide suggestions and comments on the tool. Finally, we have run three training workshops (in Bucharest, Bangkok, and Pretoria) as part of the World Bank’s 2018 Skills Building Program (themed “Big Data, Artificial Intelligence and Decision Science in Health and Nutrition”), where we trained approximately 100 users (predominantly representatives from ministries of health, development agencies, and local academic institutions).

There are several limitations to the Cascade Analysis Tool as it currently stands. Firstly, the web application was designed specifically for supporting cascade analyses, but the underlying model (Atomica) has additional functionalities that have not been introduced to the web application. For example, with Atomica one can specify a much broader range of optimization objectives (e.g. minimizing new infections, disease-related deaths, or DALYs), whereas the Cascade Analysis Tool web application only supports cascade-related objectives. Therefore, whilst it is possible to specify any compartmental model in the Cascade Analysis Tool (e.g. an SIR model with onward transmission), the set of analyses that can be conducted are limited to the cascade-related ones described in this paper. Secondly, the data requirements for running an analysis with the tool can be burdensome, especially with regards to intervention-related data on unit costs and coverage. In early pilot studies with the tool, we have found that there are increasing efforts to obtain these types of data, but they may not yet be readily available, or they may only be available for single points in time (in which case, the Cascade Analysis Tool operates under the assumption that these values are constant over time, which may not be realistic). Thirdly, the tool does not currently support discounting, so any discounting of budgets or health outcomes must be done outside of the tool. Similarly, the tool does not calculate potential resource savings, e.g. if improving treatment control outcomes leads to savings in the costs of managing disease complications; this type of calculation would also need to be done as a supplementary analysis if desired. Fourthly, initial feedback on the tool has indicated that it demands a high degree of technical sophistication and understanding of data and modelling to work as intended. We are working on iterations of the software that promote usability. We are also working on extensions to the underlying model to support new types of policy questions, including questions around equity (e.g., which interventions should be prioritized to maximize equity of access to interventions like vaccines), geographical prioritization, and interrelated diseases (e.g., prioritization of integrated services for HIV/TB).

Whilst the Cascade Analysis Tool is still in the early stages of existence, we believe there is potential for it to help make sense of the increasing quantities of data on cascades. Furthermore, we hope that the existence of this tool will help motivate the collection of even more data, so that results-based evaluation can continue to guide the decision-making processes in health and development in the future.

## Data availability

All data underlying the results are available as part of the article and no additional source data are required.

## Software availability

- Software available at: http://ui.cascade.tools/
- Source code available from: https://github.com/atomicateam/atomica
- Archived source code at time of publication: https://doi.org/10.5281/zenodo.3137154 ^30^
- License: GNU General Public License v3.0
