# Supplementary material for: The Cascade Analysis Tool: software to analyze and optimize care cascades
Source: Gates Open Res. 2019 Dec 24;3:1488. Originally published 2019 Jun 7. [Version 2] doi: 10.12688/gatesopenres.13031.2 (PMC6944813; doi:10.12688/gatesopenres.13031.2)
Supplement: Peer review history

# Peer review: Reviewer response for version 2

**DOI:** 10.21956/gatesopenres.14247.r28400

**Published:** 2020-01-16

**Authors:** Noah Haber

**Affiliations:** 1 Carolina Population Center, University of North Carolina at Chapel Hill, Chapel Hill, NC, USA; 2 Meta-Research Innovation Center, Stanford University, Stanford, CA, USA

I have re-read the document, and believe that the authors have greatly improved the paper all around. This is an excellent effort. I look forward to seeing future iterations of the concepts and software presented by this author team.

I confirm that I have read this submission and believe that I have an appropriate level of expertise to confirm that it is of an acceptable scientific standard.

# Peer review: Reviewer response for version 2

**DOI:** 10.21956/gatesopenres.14247.r28401

**Published:** 2020-01-07

**Authors:** Ramnath Subbaraman

**Affiliations:** 1 Department of Public Health and Community Medicine, Tufts University School of Medicine, Boston, MA, USA

The reviewers have addressed the primary feedback I provided, which was to provide feedback mechanism on the software platform they are describing. I approve the revised version of this manuscript.

I confirm that I have read this submission and believe that I have an appropriate level of expertise to confirm that it is of an acceptable scientific standard.

# Peer review: Reviewer response for version 2

**DOI:** 10.21956/gatesopenres.14247.r28399

**Published:** 2020-01-06

**Authors:** Mathieu Maheu-Giroux

**Affiliations:** 1 Department of Epidemiology, Biostatistics, and Occupational Health, McGill University, Montréal, QC, Canada

The authors have answered all my previous questions and concerns. I believe that the paper has been much improved.

I confirm that I have read this submission and believe that I have an appropriate level of expertise to confirm that it is of an acceptable scientific standard.

# Peer review: Reviewer response for version 1

**DOI:** 10.21956/gatesopenres.14141.r27542

**Published:** 2019-07-25

**Authors:** Mathieu Maheu-Giroux

**Affiliations:** 1 Department of Epidemiology, Biostatistics, and Occupational Health, McGill University, Montréal, QC, Canada

The paper by Kedziora and colleagues present a new software, christened “ *The Cascade Analysis Tool*”, that enables users to optimize resources allocation under budget constraints. The authors provide a generic flexible framework to model different types of cascades, applicable to a wide range of health conditions.

 The introduction is well-written and provides a nice background to explain the relevance of the *Cascade Analysis Tool*. Overall, this is a nice paper that addresses an important issue. However, I found that the methods section is (at time) difficult to read and that some relevant information is missing. These points are further detailed below.

**Suggestions:** 

1. The authors could consider adding a table or box that would summarize the key data requirements to use this tool. I understand that this can be challenging given that the tool is intended to be generic but it would inform on the key elements required. For example, do we need mortality (or e0) and disease-specific mortality rates? Do we need to have precise estimates on the number (or %) of individuals in each compartment of the cascade?
2. In the case of infectious diseases where interventions can have externalities, how is onward transmission taken into account? From my understanding of the paper, there is no force of infection specified in the compartmental model. I am not saying that it should absolutely be considered but, at the very least, the assumption of no externalities (i.e., averting chains of transmission) should be made crystal clear in the paper and the implications on budget allocation discussed, including limitations.
3. It is unclear what are the types of outcomes that can be optimized? From the case studies on the website referred to in the paper (HIV in South Africa; https://cascade.tools/south-africa), it seems that it can be “infection averted” but this is not mentioned in the paper. Where the cascade results plugged in another model (e.g., Optima) to obtain this result?
4. Also, can QALY or DALY be optimized instead? Based on the sentence “ *It is possible for users to construct their own objective using any of the model’s outputs, as well as to combine multiple objectives into a single target*”, this seems theoretically possible. In any case, I suggest being a little bit more specific about what type of objective functions can, and cannot, be optimized.
5. For resources allocation, what is the recommended time horizon for the economic evaluation/optimization? I suspect that this is case-specific and must be chosen by the users. I recommend making this clear in the paper. Similar issue with discounting? Can health outcomes and/or budgets be discounted at a user-specified rate? I am not asking that the authors go in details about this… but if these functionalities are readily available (or not) should be mentioned in the relevant section(s).
6. Also regarding resources allocation, can potential resources savings be considered? For example, earlier management of a chronic condition could save money down the line and this could impact decision-making regarding best allocative strategies.
7. The paper does an excellent job at explaining which type of questions can be answered with the *Cascade Analysis Tool*. It would be equally informative if examples of policy questions that cannot be answered (or with difficulty) were presented. For example, the optimization algorithm seems to be agnostic about equity constraints (is that the case)? Can resources allocation among cascades in different geographical regions be performed? Can optimizing two cascades (for different health conditions) that could share some common interventions be achieved?
8. Figure 5 is confusing as the “ *LTFU rate*”, “ *Control rate*”, and “ *Death rate*” are depicted as unit-less probability. Are these rates (incidence density) or proportions (incidence proportion)?
9. Can the discussion of the limitations of the model be expanded. For example, what are the implications of the steady state assumption? How much training is needed for public health practitioners to be able to perform analyses on their own with the *Cascade Analysis Tool*? What set of minimum core competencies are required?

 
**Discretionary revisions** 

1. The Methods section has many different types of headers without any clear ordering. Please standardize all headers and sub-headers as the current format is confusing.
2. Why start the methods with the different packages used ( *Implementation* section)? I am not convinced that this is relevant to your audience.
3. “ *Characteristics*” is confusing to described quantities that can be derived from the model? Maybe “ *Indicators*” or “ *Derived metrics*” would be more appropriate?
4. Is it really the “ *summed loss rates*” that are being minimized or the total number of individuals lost at all stages? It seems that these two different ways of conceptualizing losses could give different results.
5. Why provide a link to the software page if it is password-protected? Will a fee be charged to use it?
6. Consider renaming the section “ *Use cases*” to “ *Case studies*”.
7. The GitHub repo refers to “ *Atomica*”. It seems that the latter include more features than the *Cascade Analysis Tool*, which is slightly confusing. Also, the *readme* of this repo does not mention the *Cascade Analysis Tool*. Are “ *Atomica*” and the “ *Cascade Analysis Tool*” the same? If so, could the repo be renamed.

I confirm that I have read this submission and believe that I have an appropriate level of expertise to confirm that it is of an acceptable scientific standard, however I have significant reservations, as outlined above.

# Response

**Published:** 2020-01-11

**Authors:** Robyn Stuart

**Affiliations:** University of Copenhagen, Denmark

We thank the reviewer for taking the time to review, and for providing helpful comments that have greatly strengthened the article. Responses to particular points are provided below.

- The paper by Kedziora and colleagues present a new software, christened “The Cascade Analysis Tool”, that enables users to optimize resources allocation under budget constraints. The authors provide a generic flexible framework to model different types of cascades, applicable to a wide range of health conditions. **We thank the reviewer for the summary.**
- The introduction is well-written and provides a nice background to explain the relevance of the Cascade Analysis Tool. Overall, this is a nice paper that addresses an important issue. However, I found that the methods section is (at time) difficult to read and that some relevant information is missing. **Thank you, we have reworked the methods section considerably in response to this and other reviewer comments - details are provided below.**
- The authors could consider adding a table or box that would summarize the key data requirements to use this tool. I understand that this can be challenging given that the tool is intended to be generic but it would inform on the key elements required. For example, do we need mortality (or e0) and disease-specific mortality rates? Do we need to have precise estimates on the number (or %) of individuals in each compartment of the cascade? **We have added a summary paragraph on key data requirements to the methods section, as well as a link to the section of the website where this information is summarized.**
- In the case of infectious diseases where interventions can have externalities, how is onward transmission taken into account? From my understanding of the paper, there is no force of infection specified in the compartmental model. I am not saying that it should absolutely be considered but, at the very least, the assumption of no externalities (i.e., averting chains of transmission) should be made crystal clear in the paper and the implications on budget allocation discussed, including limitations. **Atomica, the model that powers the Cascade Analysis Tool, can be used to set up arbitrary compartmental models, including models with a force of infection and onward transmission. This functionality is carried over to the Cascade Analysis Tool, but it’s not the main intended use case of the tool. We have tried to clarify this in the discussion section. **
- It is unclear what are the types of outcomes that can be optimized? From the case studies on the website referred to in the paper (HIV in South Africa; https://cascade.tools/south-africa), it seems that it can be “infection averted” but this is not mentioned in the paper. Where the cascade results plugged in another model (e.g., Optima) to obtain this result? **We have added text to the limitations paragraph of the discussion section on this: “the web application was designed specifically for supporting cascade analyses, but the underlying model (Atomica) has additional functionalities that have not been introduced to the web application. For example, with Atomica one can specify a much broader range of optimization objectives (e.g. minimizing new infections, disease-related deaths, or DALYs), whereas the Cascade Analysis Tool web application only supports cascade-related objectives. Therefore, whilst it is possible to specify any compartmental model in the Cascade Analysis Tool (e.g. an SIR model with onward transmission), the set of analyses that can be conducted in the web application are limited to the cascade-related ones described in this paper.”**
- Also, can QALY or DALY be optimized instead? Based on the sentence “It is possible for users to construct their own objective using any of the model’s outputs, as well as to combine multiple objectives into a single target”, this seems theoretically possible. In any case, I suggest being a little bit more specific about what type of objective functions can, and cannot, be optimized.**** **Yes, see above.**
- For resources allocation, what is the recommended time horizon for the economic evaluation/optimization? I suspect that this is case-specific and must be chosen by the users. I recommend making this clear in the paper. Similar issue with discounting? Can health outcomes and/or budgets be discounted at a user-specified rate? I am not asking that the authors go in details about this… but if these functionalities are readily available (or not) should be mentioned in the relevant section(s). **Indeed, this is all to be specified by the user. Discounting is not currently supported, and we have added a note on this to the limitations section. **
- Also regarding resources allocation, can potential resources savings be considered? For example, earlier management of a chronic condition could save money down the line and this could impact decision-making regarding best allocative strategies. **This is not possible, we’ve added this to the limitations. **
- The paper does an excellent job at explaining which type of questions can be answered with the Cascade Analysis Tool. It would be equally informative if examples of policy questions that cannot be answered (or with difficulty) were presented. For example, the optimization algorithm seems to be agnostic about equity constraints (is that the case)? Can resources allocation among cascades in different geographical regions be performed? Can optimizing two cascades (for different health conditions) that could share some common interventions be achieved? **We’ve added some text addressing this point to the discussion section: “We are also working on extensions to the underlying model to support new types of policy questions, including questions around equity (e.g., which interventions should be prioritized to maximize equity of access to interventions like vaccines), geographical prioritization, and interrelated diseases (e.g., prioritization of integrated services for HIV/TB).”**
- **Figure 5 is confusing as the “LTFU rate”, “Control rate”, and “Death rate” are depicted as unit-less probability. Are these rates (incidence density) or proportions (incidence proportion)? **We have addressed this (also noted by reviewer 1). 
- Can the discussion of the limitations of the model be expanded. For example, what are the implications of the steady state assumption? How much training is needed for public health practitioners to be able to perform analyses on their own with the Cascade Analysis Tool? What set of minimum core competencies are required? **We have significantly expanded the section on limitations and addressed these points. **
- The Methods section has many different types of headers without any clear ordering. Please standardize all headers and sub-headers as the current format is confusing.We have rearranged this section (also in response to comments from the other two reviewers). Why start the methods with the different packages used (Implementation section)? I am not convinced that this is relevant to your audience.**** **According to the journal submission guidelines, this type of article needs to include subsections on Implementation (describing how the tool works and any relevant technical details required for implementation) and Operation (including the minimal system requirements needed to run the software and an overview of the workflow). This subsection on which packages were used was added as per an editor request.**
- “Characteristics” is confusing to described quantities that can be derived from the model? Maybe “Indicators” or “Derived metrics” would be more appropriate? **We agree with this suggestion and have removed the word “characteristics”. **
- Is it really the “summed loss rates” that are being minimized or the total number of individuals lost at all stages? It seems that these two different ways of conceptualizing losses could give different results. **We have changed to: “Minimize the total number of people lost from each stage of the cascade”.**
- Why provide a link to the software page if it is password-protected? Will a fee be charged to use it? **The software requires users to log in, but it is free to create an account. New users can register by clicking “Register here” at http://ui.cascade.tools/**
- **Consider renaming the section “Use cases” to “Case studies”.**“Use cases” is suggested by the article submission guidelines. 
- The GitHub repo refers to “Atomica”. It seems that the latter include more features than the Cascade Analysis Tool, which is slightly confusing. Also, the readme of this repo does not mention the Cascade Analysis Tool. Are “Atomica” and the “Cascade Analysis Tool” the same? If so, could the repo be renamed. **We agree it was confusing and have clarified in the text. **

# Peer review: Reviewer response for version 1

**DOI:** 10.21956/gatesopenres.14141.r27363

**Published:** 2019-07-22

**Authors:** Ramnath Subbaraman

**Affiliations:** 1 Department of Public Health and Community Medicine, Tufts University School of Medicine, Boston, MA, USA

This is a very thoughtfully written article on a potentially helpful software tool. The goal of the tool is: 

1. To facilitate the construction of care cascades for different diseases, including a flexible platform for developing such care cascades that recognizes that each disease will have different steps/stages required and different approaches to patient transitions between stages.
2. Facilitate construction of care cascade by specific sub-populations (e.g., gender, presumably age, etc.)
3. To allow estimation of the impact of interventions to improve outcomes in disease-specific care cascades.

Overall, the manuscript is very well-written and communicates key points in sufficient detail. More sophisticated critiques would emerge only, I think, as more people practically use this software for programmatic purposes. 

 I have the following major and minor points of feedback:

 Major Feedback:

1. Ideally, there would be some platform for public comment, critique, and feedback on this software platform as users engage with it. One of the challenges with reviewing this manuscript is that, while I spent a bit of time playing with the software, it is challenging to provide specific feedback on its strengths or deficiencies without actually using it in practice for a specific disease and programmatic analysis. As such, I think ongoing public feedback on the software from actual users is more critical than peer review of this manuscript.
2. Care cascades for different diseases deal with transitions differently. For example, most HIV care cascades look at transitions backwards as well as forwards across stages (or "compartments"). In contrast, in most care cascades for active TB, patients can only move forward across care cascade stages, and they can have multiple types of poor outcomes in each "gap" (i.e., difference between steps / stages / compartments). For example, for patients who start treatment but do not complete TB treatment, they could have one of 3 poor outcomes: (a) death, (b) treatment failure, and (c) loss to follow-up. The assumption is that "loss to follow-up" patients do not move "backwards" in the care cascade but fall out completely (and would have to restart from the beginning). Two questions related to this: (a) can this software modeling approach manage care cascades that only have transitions in the forward direction (I am assuming yes from Fig 3, but would be helpful to clarify); and (b) can this software modeling approach capture and estimate different types of poor outcomes as described above? 

Clarifying both of these questions in the text would be helpful for readers coming from different disease backgrounds and envisioning the approach differently. 

 A key point in the manuscript is this statement: "In many real-world situations, the impact of changing intervention coverage and the way interventions should be prioritized is not clear from the analysis of intervention properties alone." This is an important point that may not be evident to readers who don't frequently look at modeling data that look at broader implications and impacts of interventions. When I read this, I had a few thoughts regarding how the authors could expand on this point:

1. It would be helpful for the authors to add a few sentences explaining this point further. If we already have data on a particular intervention and its impact on reducing a given gap in care, what are the additional benefits of the type of modeling proposed by the authors? 
2. One practical problem that users of this software will face may be the lack of available data on the effect sizes and costing of different interventions to reduce gaps in care. It would be helpful for the authors to specify what types of data / findings users of the software should have available to use this tool. Do they need effect sizes of an intervention to reduce a gap in the care cascade (i.e., estimated % reduction in a poor outcome at a particular step from an intervention)? Do they need confidence intervals for this effect estimate? Do they need these effect size estimates for an intervention for multiple care cascade steps (if an intervention improves outcomes at multiple steps)? What kind of costing data do they need to use this tool? 

Finally, it would be helpful for the authors to provide some real world examples of the types of studies that are available from which users of the software can provide these estimates. Even providing citations, example effect sizes or costing data from the existing literature for studies from HIV (for example, since they are most broadly available here) would help readers to think about the types of data they can seek out for their own diseases to populate these models.

 Minor Comments:

1. Authors might consider including a citation for the Lancet Global Health Commission on High-Quality Health Systems, as that major report has argued for care cascade analyses as being a central component of health quality dashboards for understanding quality of care. This strengthens the argument for a user friendly tool such as the one created by these authors.
2. In paragraph 2 of the paper, the authors should also note that the phrase "continuum of care" has also been used for these types of analyses. This is now emerging as the predominant language for these analyses in HIV, with the assumption that patients can move backwards as well as forwards along this continuum of care.
3.  Paragraph 3 of the Introduction: The authors can correct the way they have described the TB care cascade analyses. The India and South Africa care cascades that have been cited for TB look at active TB disease, while the latent TB care cascade cited looks at latent TB disease. As such, these are two different frameworks for different types of TB--i.e., it is not that the framework in citation 10 guides the framework in citations 11 and 12--they are different frameworks for different forms of TB. A more detailed methods framework for active TB disease is available in the manuscript: Subbaraman R, Nathavitharana RR, Mayer KH, Satyanarayana S, Chadha VK, Arinaminpathy N, et al. (2019) Constructing care cascades for active tuberculosis: A strategy for program monitoring and identifying gaps in quality of care ^1^. Such methodological manuscripts could inform development of models using a tool such as the one the authors have created.

I confirm that I have read this submission and believe that I have an appropriate level of expertise to confirm that it is of an acceptable scientific standard, however I have significant reservations, as outlined above.

## References

[1] : Constructing care cascades for active tuberculosis: A strategy for program monitoring and identifying gaps in quality of care. *PLoS Med*.2019;16(2) : 10.1371/journal.pmed.1002754 e1002754 10.1371/journal.pmed.1002754 30811385PMC6392267

# Response

**Published:** 2020-01-11

**Authors:** Robyn Stuart

**Affiliations:** University of Copenhagen, Denmark

- Ideally, there would be some platform for public comment, critique, and feedback on this software platform as users engage with it. One of the challenges with reviewing this manuscript is that, while I spent a bit of time playing with the software, it is challenging to provide specific feedback on its strengths or deficiencies without actually using it in practice for a specific disease and programmatic analysis. As such, I think ongoing public feedback on the software from actual users is more critical than peer review of this manuscript.**** **We thank the reviewer for this excellent suggestion. We have added a blog to our website (where we can also post information on updates to the tool). Users can provide feedback via the comments section. **
- Care cascades for different diseases deal with transitions differently. For example, most HIV care cascades look at transitions backwards as well as forwards across stages (or "compartments"). In contrast, in most care cascades for active TB, patients can only move forward across care cascade stages, and they can have multiple types of poor outcomes in each "gap" (i.e., difference between steps / stages / compartments). For example, for patients who start treatment but do not complete TB treatment, they could have one of 3 poor outcomes: (a) death, (b) treatment failure, and (c) loss to follow-up. The assumption is that "loss to follow-up" patients do not move "backwards" in the care cascade but fall out completely (and would have to restart from the beginning). Two questions related to this: (a) can this software modeling approach manage care cascades that only have transitions in the forward direction (I am assuming yes from Fig 3, but would be helpful to clarify); and (b) can this software modeling approach capture and estimate different types of poor outcomes as described above? Clarifying both of these questions in the text would be helpful for readers coming from different disease backgrounds and envisioning the approach differently.**** **Yes, both types of cascade can be modelled with the tool. We have added text on this to the methods section.**
- A key point in the manuscript is this statement: "In many real-world situations, the impact of changing intervention coverage and the way interventions should be prioritized is not clear from the analysis of intervention properties alone." This is an important point that may not be evident to readers who don't frequently look at modeling data that look at broader implications and impacts of interventions. When I read this, I had a few thoughts regarding how the authors could expand on this point:
  - It would be helpful for the authors to add a few sentences explaining this point further. If we already have data on a particular intervention and its impact on reducing a given gap in care, what are the additional benefits of the type of modeling proposed by the authors? 
  - One practical problem that users of this software will face may be the lack of available data on the effect sizes and costing of different interventions to reduce gaps in care. It would be helpful for the authors to specify what types of data / findings users of the software should have available to use this tool. Do they need effect sizes of an intervention to reduce a gap in the care cascade (i.e., estimated % reduction in a poor outcome at a particular step from an intervention)? Do they need confidence intervals for this effect estimate? Do they need these effect size estimates for an intervention for multiple care cascade steps (if an intervention improves outcomes at multiple steps)? What kind of costing data do they need to use this tool? 
  - Finally, it would be helpful for the authors to provide some real world examples of the types of studies that are available from which users of the software can provide these estimates. Even providing citations, example effect sizes or costing data from the existing literature for studies from HIV (for example, since they are most broadly available here) would help readers to think about the types of data they can seek out for their own diseases to populate these models.
- **We have:**
  - **Moved this text to a more prominent position in the article, and emphasized it more clearly by repeating it in the discussion section as well. **
  - **“One practical problem that users of this software will face may be the lack of available data on the effect sizes and costing of different interventions to reduce gaps in care.” -- we added a section noting the issues related to data availability in the methods. **
  - **“It would be helpful for the authors to specify what types of data / findings users of the software should have available to use this tool.” - we added a section on minimum data requirements to the data entry section of the methods**
  - **“Do they need effect sizes of an intervention to reduce a gap in the care cascade (i.e., estimated % reduction in a poor outcome at a particular step from an intervention)” -- yes, this is needed, and we’ve added text clarifying this.**
  - **“Do they need confidence intervals for this effect estimate?” -- this is not required.**
  - **“Do they need these effect size estimates for an intervention for multiple care cascade steps (if an intervention improves outcomes at multiple steps)? What kind of costing data do they need to use this tool?” - we have specified this in the data entry section.**
  - **We have added a paragraph on typical data sources.**
- Authors might consider including a citation for the Lancet Global Health Commission on High-Quality Health Systems, as that major report has argued for care cascade analyses as being a central component of health quality dashboards for understanding quality of care. This strengthens the argument for a user friendly tool such as the one created by these authors. **Thank you, we have added this.**
- In paragraph 2 of the paper, the authors should also note that the phrase "continuum of care" has also been used for these types of analyses. This is now emerging as the predominant language for these analyses in HIV, with the assumption that patients can move backwards as well as forwards along this continuum of care. **Thank you, we have added this.**
- Paragraph 3 of the Introduction: The authors can correct the way they have described the TB care cascade analyses. The India and South Africa care cascades that have been cited for TB look at active TB disease, while the latent TB care cascade cited looks at latent TB disease. As such, these are two different frameworks for different types of TB--i.e., it is not that the framework in citation 10 guides the framework in citations 11 and 12--they are different frameworks for different forms of TB. A more detailed methods framework for active TB disease is available in the manuscript: Subbaraman R, Nathavitharana RR, Mayer KH, Satyanarayana S, Chadha VK, Arinaminpathy N, et al. (2019) Constructing care cascades for active tuberculosis: A strategy for program monitoring and identifying gaps in quality of care1. Such methodological manuscripts could inform development of models using a tool such as the one the authors have created. **Thank you, we have fixed this and added the suggested citation.**

# Peer review: Reviewer response for version 1

**DOI:** 10.21956/gatesopenres.14141.r27336

**Published:** 2019-06-25

**Authors:** Noah Haber

**Affiliations:** 1 Carolina Population Center, University of North Carolina at Chapel Hill, Chapel Hill, NC, USA; 2 Meta-Research Innovation Center, Stanford University, Stanford, CA, USA

**General comments**

 I believe that this software appears to be a strong and useful effort toward making useful decisions based on cascades, under a generalized framework. The authors have identified an important problem, and have made substantial progress towards providing a useful solution. As a preliminary and experimental release, I believe this to be a successful effort. However, some caveats should be considered with regard to the software and its accompanying paper as they currently stand.

 A general concern is that software is requires a high degree of sophistication and understanding of data and modelling to work as intended. The effort and sophistication required is highlighted in the example, where the assumptions required are often very serious, and highly consequential.

 In addition, the report as it stands could be more clearly organized and give additional technical detail in the methods section.

 As the authors note, it shows promise and potential. Getting it from a proof of concept that solves very interesting problems to being useful/usable to relatively novice users is long process. I really hope that this effort continues to develop into maturity.

**Methods**

 General comments: I would suggest a fairly substantial re-organization for this section. Quite a bit of time is spent on introducing people to the concept of a cascade (which is not the innovative part of this paper), at the expense of what is useful, interesting, and innovative about this tool.

 The section reads like a manual for designing a cascade analysis with the tool, rather than an explanation of what the software actually does and the methods and math underlying it. Quite a few sections are not the software methods at all, and should be moved to the introduction or removed. I am not sure I would be able to recreate a near equivalent of this tool without much more detail on the underlying model itself.

 I found that I did not understand how the model actually functioned, beyond that it was a compartmental model. Showing some of the math a bit more (even as an example, or just citations) would go a long way toward helping understand what's going on.

*Designing the cascade*

 I think the language used for this framework could use a major revisit. It took me quite a bit of time and going backward to figure out what the authors were referring to throughout, which was distracting for the later sections.

 In the context of the user-oriented manual view, referring to the stages as "compartments" seems out of place and unnecessary, when "stage" or "state" seems more appropriate and more general for users. If this is an explanation of the methods of the model, then saying it is based on a compartmental model (and specifying exactly how said model is parameterized) is useful.

 "Parameter" has a much broader meaning, and forcing "parameter" to mean this very specific thing, is confusing. I would suggest referring to this as "transition properties" or "transition parameters" and collapse this paragraph with the previous. Also, what, specifically are the required parameters, and in what units? At minimum, transition rates need to be entered, correct?

 The same applies to the word "characteristics". Why not just use "stage groups" or similar?

*Collecting data*

 Again, a bit strange here. Data entry and data collection are two different things. Which are the authors trying to describe, and do they belong in the methods or in the use example? As an aside, I find the Excel interface bit difficult to use, and unintuitive why I would have to exit the site to enter data. It would be far more straightforward to have a browser-based UI for this, with optional support for Excel-based data entry.

*Analysis of policy questions*

 I believe that this belongs in the intro.

*Cascade optimization*

 This is where the good stuff is! Lead with this, shorten/consolidate/remove the rest. I found this section to be relatively clear and well-written, but unfortunately buried underneath everything else.

**Example cascade**

 One thing this section really highlights is the incredible amount of sophisticated work users need to do to make all this work together. Possibly to the point that a user who is able to adequately generate these numbers could very nearly model this all themselves. This is a first major effort, so that is to be expected, but it should be considered strongly.

 It would be helpful if this was more obviously linked to the compartments. Example: We estimated the transition rate from stage X to stage Y by ____. 

*"We further assume that the hypertension cascade is in a steady state such that the relative proportions of people in each cascade stage are constant over time, which implies that the number of people in each cascade stage will increase by 4.4% each year."*

 This is a suspect and (as far as I can tell) unnecessary assumption. If this were true, there would be no change in the proportions between years (which there are, as shown in Figure 4). Why not use between-year flow rates (noting a few minor assumptions)? I might suggest that adding a mini model to solve for those flow rates would be an extremely useful feature, as forcing users to do this on their own will be error-prone. While is just an example, but it's a potentially distracting error.

**Discussion**

 I would suggest that the discussion section have a much more comprehensive treatment of limitations of the tool. What kinds of things can it not model? Am I able to create a fully fledged SIR model, for example? That might be implied by the use of compartment modelling, it isn't clear to me whether or not this is possible.

 One clear limitation of this model is that it has a bit of a strange relationship with time. A slightly pedantic issue is that the authors appear to be using rates and probabilities interchangeably. More importantly, enormous assumptions must be made in order to turn the cross-sectional data sources into what appears to be models over time. Issues in these assumptions and pre-model modelling can lead to severe misallocation of resources.

 I believe this to be a useful tool, albeit one that would benefit greatly from real-world testing, and I hope that the authors are able to continue developing it.

I confirm that I have read this submission and believe that I have an appropriate level of expertise to confirm that it is of an acceptable scientific standard, however I have significant reservations, as outlined above.

# Response

**Published:** 2020-01-11

**Authors:** Robyn Stuart

**Affiliations:** University of Copenhagen, Denmark

- I believe that this software appears to be a strong and useful effort toward making useful decisions based on cascades, under a generalized framework. The authors have identified an important problem, and have made substantial progress towards providing a useful solution. As a preliminary and experimental release, I believe this to be a successful effort. However, some caveats should be considered with regard to the software and its accompanying paper as they currently stand. **We thank the reviewer for the summary.**
- A general concern is that software requires a high degree of sophistication and understanding of data and modelling to work as intended. The effort and sophistication required is highlighted in the example, where the assumptions required are often very serious, and highly consequential. **This is well noted, and in response to this comment we have (a) revised the manuscript so it caters to a less technically knowledgeable audience, and (b) added text summarizing these concerns raised by the reviewer to the limitations paragraph of the discussion section. **
- In addition, the report as it stands could be more clearly organized and give additional technical detail in the methods section. **We have attempted to streamline and clarify the methods section (also in response to comments from the other reviewers). **
- As the authors note, it shows promise and potential. Getting it from a proof of concept that solves very interesting problems to being useful/usable to relatively novice users is a long process. I really hope that this effort continues to develop into maturity. **We thank the reviewer for the kind words and well wishes. As per a comment from the second reviewer, we have established a mechanism for gathering user feedback. We are looking forward to further improving our software in response to user comments.**
- Methods General comments: I would suggest a fairly substantial re-organization for this section. Quite a bit of time is spent on introducing people to the concept of a cascade (which is not the innovative part of this paper), at the expense of what is useful, interesting, and innovative about this tool. The section reads like a manual for designing a cascade analysis with the tool, rather than an explanation of what the software actually does and the methods and math underlying it. Quite a few sections are not the software methods at all, and should be moved to the introduction or removed. I am not sure I would be able to recreate a near equivalent of this tool without much more detail on the underlying model itself. I found that I did not understand how the model actually functioned, beyond that it was a compartmental model. Showing some of the math a bit more (even as an example, or just citations) would go a long way toward helping understand what's going on.We have rearranged this section significantly. **Firstly, we have expanded the section on implementation (NB - the section headings are taken from the article guidelines on how software tool articles should be structured). The reviewer makes a good point that we were previously lacking detail on how the software is actually structured, so we have added this to the implementation section. Creating a similar tool would be possible by following the architecture in Figure 3.** **Much of the math behind the model is actually handled by the Atomica package, and we don’t go into detail about it here. However, we’ve added more detail in the example. **
- Designing the cascade: I think the language used for this framework could use a major revisit. It took me quite a bit of time and going backward to figure out what the authors were referring to throughout, which was distracting for the later sections. In the context of the user-oriented manual view, referring to the stages as "compartments" seems out of place and unnecessary, when "stage" or "state" seems more appropriate and more general for users. If this is an explanation of the methods of the model, then saying it is based on a compartmental model (and specifying exactly how said model is parameterized) is useful. "Parameter" has a much broader meaning, and forcing "parameter" to mean this very specific thing, is confusing. I would suggest referring to this as "transition properties" or "transition parameters" and collapse this paragraph with the previous. Also, what, specifically are the required parameters, and in what units? At minimum, transition rates need to be entered, correct? The same applies to the word "characteristics". Why not just use "stage groups" or similar? **We have reworded this section (also noted in the comments from the two other reviewers). We wish to emphasize the connection between the cascade modelling methodology and compartmental models, so we have retained “compartment” and “transition”. We have also retained “parameter”, even though we agree it has a very broad meaning, because it is heavily entwined in the software itself and is used with internal consistency in this article. However, we have removed “characteristics” (also noted by another reviewer), and we have written a paragraph on the minimal data requirements. We intend for this section to help readers understand the relationship between a compartmental model and a cascade representation. **
- Collecting data: Again, a bit strange here. Data entry and data collection are two different things. Which are the authors trying to describe, and do they belong in the methods or in the use example? As an aside, I find the Excel interface bit difficult to use, and unintuitive why I would have to exit the site to enter data. It would be far more straightforward to have a browser-based UI for this, with optional support for Excel-based data entry.**** **We renamed this section “Data entry”.** **We experimented with the idea of having users enter data via the web application itself, but experience with users led us to understand that people are very accustomed to entering data via Excel, and that doing so allows them to more easily share files with colleagues and collaborate effectively. In the end, we decided to offer Excel support since that’s what most users seemed to want. **
- Analysis of policy questions: I believe that this belongs in the intro.**** **We have shifted this to the intro.**
- Cascade optimization: This is where the good stuff is! Lead with this, shorten/consolidate/remove the rest. I found this section to be relatively clear and well-written, but unfortunately buried underneath everything else.**** **We have shortened the rest of the “Operation” section of the methods (again, this is a required section heading for this article type), so that this is placed more prominently in the methods.**
- Example cascade: One thing this section really highlights is the incredible amount of sophisticated work users need to do to make all this work together. Possibly to the point that a user who is able to adequately generate these numbers could very nearly model this all themselves. This is a first major effort, so that is to be expected, but it should be considered strongly. It would be helpful if this was more obviously linked to the compartments. Example: We estimated the transition rate from stage X to stage Y by ____. **In our experience, it is not uncommon for people to have access to all the data summarized in this example section, but not to know how to combine it and analyze it to get policy recommendations.**
- "We further assume that the hypertension cascade is in a steady state such that the relative proportions of people in each cascade stage are constant over time, which implies that the number of people in each cascade stage will increase by 4.4% each year." This is a suspect and (as far as I can tell) unnecessary assumption. If this were true, there would be no change in the proportions between years (which there are, as shown in Figure 4). Why not use between-year flow rates (noting a few minor assumptions)? I might suggest that adding a mini model to solve for those flow rates would be an extremely useful feature, as forcing users to do this on their own will be error-prone. While is just an example, but it's a potentially distracting error. **We have removed this.**
- Discussion: I would suggest that the discussion section have a much more comprehensive treatment of limitations of the tool. What kinds of things can it not model? Am I able to create a fully fledged SIR model, for example? That might be implied by the use of compartment modelling, it isn't clear to me whether or not this is possible. **We have added a paragraph on limitations.**
- One clear limitation of this model is that it has a bit of a strange relationship with time. A slightly pedantic issue is that the authors appear to be using rates and probabilities interchangeably. More importantly, enormous assumptions must be made in order to turn the cross-sectional data sources into what appears to be models over time. Issues in these assumptions and pre-model modelling can lead to severe misallocation of resources.**** **It’s a very good point (not pedantic!) about rates vs probabilities - we have clarified this throughout. We have also added text in the limitations paragraph of the discussion section on the potential issues related to using single time-points as data sources for inform time-dependent estimates. **
- I believe this to be a useful tool, albeit one that would benefit greatly from real-world testing, and I hope that the authors are able to continue developing it. **Thank you for taking the time to review!**
